# Effects of Bergamot (*Citrus bergamia* Risso) By-Product on Growth Performance and Meat Quality of Growing Rabbits

**DOI:** 10.3390/foods13162611

**Published:** 2024-08-20

**Authors:** Manuel Scerra, Francesco Foti, Pasquale Caparra, Matteo Bognanno, Paolo Fortugno, Domenico Autolitano, Domenico Viglianti, Marco Sebastiano Bella, Marco Sebastiano Cannone, Luigi Chies

**Affiliations:** 1 Produzioni Animali Unit, Dipartimento di Agraria, University of Reggio Calabria, Via dell’Università 25, 89124 Reggio Calabria, Italy; francesco.foti@unirc.it (F.F.); pasquale.caparra@unirc.it (P.C.); matteo.bognanno@unirc.it (M.B.); frtpla99s23h224h@studenti.unirc.it (P.F.); domenico2405@icloud.com (D.A.); vgldnc01b15f112u@studenti.unirc.it (D.V.); lchies@unirc.it (L.C.); 2 Dipartimento di Agricoltura, Alimentazione e Ambiente (Di3A), University of Catania, Via Valdisavoia 5, 95123 Catania, Italy; marco.bella@unict.it (M.S.B.); marco.cannone@unict.it (M.S.C.)

**Keywords:** antioxidant, fatty acid composition, oxidative stability, phenols, shelf-life

## Abstract

This study aimed to investigate the effects of feeding dried bergamot pulp to rabbits on animal performance and meat quality. Thirty rabbits were assigned to two groups (balanced for body weight, 804.4 ± 2.35 g) and fed individually for 60 days a basal diet (control) or the basal diet in which part of the cereals was replaced with 10% of dried bergamot pulp (DBP). There were no effects of DBP on growth performance, carcass yield, or the crude protein and ether extract composition of meat. The concentrations of α-linolenic acid (C18:3 n-3) and eicosapentaenoic acid (C20:5 n-3) increased in the *longissimus thoracis et lumborum* muscle (*p* < 0.01 and *p* = 0.021, respectively) after integrating dried bergamot pulp into the diet, leading to higher levels of total of ω-3 fatty acids (*p* < 0.01) compared to the control treatment. The inclusion of dried bergamot pulp improved the oxidative stability in meat (*p* < 0.001), where TBARS values were lower after 4 and 7 days of refrigerated storage (*p* < 0.001) in the DBP group than in the control group. Finally, feeding dried bergamot pulp to rabbits improves meat quality without negatively influencing growth performance.

## 1. Introduction

In recent years, the increases in the cost of raw materials and in general in the cost of living have inevitably led to an increase in the cost of protein sources of animal origin. In the first six months of 2023, spending on beef increased by 6.7% compared to the same period in 2022, with an overall increase of more than 20% from 2020 [1]. Therefore, never before has the re-evaluation of low-cost animal protein sources played a fundamental role. From this perspective, the rabbit industry has always played an important role in providing humans with animal proteins at low cost, a meat also appreciated for its low fat and cholesterol content [2].

Among the main ingredients used for rabbit diets are corn, alfalfa hay, and soybean meal. They are feedstuffs that particularly influence the cost of the diet as they have a high commercial value [3]. Therefore, even in the rabbit sector, the use of alternative feedstuffs to reduce meat production costs would be necessary, considering that in 2023, the price of meat increased by around 15% in the rabbit sector too.

The use of agro-industrial by-products as animal feedstuffs, in addition to reducing dietary costs, can have an important role in waste management. Among them, dried citrus pulps (DCP), a mixture of citrus peel, pulp, and seeds, have been evaluated as possible dietary ingredients in monogastric and polygastric animals with interesting results [4,5]. This by-product is characterized by a high content of readily fermentable carbohydrates and by a modest protein content [6]. Furthermore, several substances with strong antioxidant [5], such as phenols and vitamins, antibacterial [7], and immunostimulatory [8] properties are present.

In recent years, several studies on the use of bergamot (*Citrus bergamia* Risso) processing residues in sheep, goat, and pig diets have been carried out to evaluate their effects on growth performance and meat quality [9,10,11]. The interest in bergamot fruit arises from the interesting characteristics that distinguish it from other citrus fruits. Some studies report a significantly higher amount of flavonoids in the peel of bergamot fruit in contrast to other citrus peels [12,13], compounds which, above all thanks to their antioxidant activity, have shown interesting healthy properties [14]. Considering the interesting characteristics of this by-product and that, to the best of our knowledge, there are no studies in the literature that have explored the effect of bergamot by-product on the meat quality of small animals such as rabbit, it would be useful to study its effects on the quality of rabbit meat, a product that provides humans with animal protein with a high biological value at low cost. Our hypothesis was that the addition of dried bergamot pulp could improve the quality of rabbit meat, such as meat oxidative stability, without negatively affecting growth performance. Therefore, the aim of the present study was to investigate the effects of the dietary inclusion of dried bergamot pulp on the growth performance, carcass yield, and meat quality of rabbit.

## 2. Materials and Methods

The experiment was approved (prot. No. 1214) by the Animal Welfare Committee of the University of Reggio Calabria.

Thirty HyCole (5-week-old) rabbits, were allotted in two groups of 12 rabbits per treatment, balanced for body weight (804.4 ± 2.35 g) into CON (control) and DBP (dried bergamot pulp) groups, and allocated in individual wire cages (40 cm × 30 cm × 50 cm height, width, and length, respectively) supplied with separated feeders. This experimental trial was extended for 60 days. After 5 days of the adaptation period, rabbits from the CON group received a basal diet, whereas the rabbits from the DBP group received the basal diet in which 5% of the barley grain and 5% of maize grain was replaced by 10% of dried bergamot pulp (Table 1). The experimental diets were formulated to cover the nutrient requirements of growing rabbits, as recommended by NRC [15]. A local citrus juice industry (Bova, Bova Marina, RC, Italy) supplied the bergamot pulp, composed of peel, pulp, and seeds, which was dried in a ventilated oven at 42 °C to a constant weight. The chemical composition, fatty acid profile, and antioxidant compounds of the dried bergamot pulp are shown in Table 1. All feeds were supplied in pellet form, approximately 10 mm long with a diameter of 4 mm. Rabbits received their respective experimental feeds ad libitum for sixty days, besides the clean water constantly available from stainless steel nipples fixed in the cage. Rabbits were individually weighed at start the experiment and in weekly intervals up to when the feeding trial was finished (day 60 of the experimental trial) to evaluate the average daily gain (ADG). All the rabbits were fed twice daily (0800 and 1500 h). Refuses were weighed and stored as a composite sample during the experiment. Feed consumed was considered the total amount offered minus the total amount refused recorded during the entire period. Feed samples were taken to determine the DM (dry matter) content and calculate DM intake. The feed conversion ratio (FCR) was calculated as DMI/ADG.

At the end of the experiment trial, at 100 days of age (35 days of age at the start of the trial, 5 days of adaptation to the experimental treatment and 60 days of experimental trial) and an average weight of 2950 g, all the animals were slaughtered via CO_2_ after a fasting period (8 h, feed), and the carcasses were weighed after skin removal and evisceration and subsequently stored at 4 °C. After 24 h, the *longissimus thoracis et lumborum* muscle (LTL) was removed from each carcass and prepared for the analyses.

### 2.1. Feedstuff Analysis and Meat Proximate Analysis

AOAC methods [16] were used to analyze feed samples for dry matter (method 934.01), crude protein (method 984.13), crude fat (method 920.39), and ash (method 942.05) contents, while the Van Soest et al. [17] analytic method was used to evaluate the neutral detergent fiber (NDF) content. Phenolic compounds were determined in feed samples following the Folin–Ciocalteau method [18]. Feed samples were analyzed for fatty acid composition following the method proposed by Gray et al. [19].

Liposoluble vitamins were determined in 200 mg of feed samples [20] treated with 3 mL of a 1:1:1 (*v*:*v*:*v*) methanol/acetone/petroleum ether solution. Then, the supernatant was evaporated and the residues obtained were dissolved in 1 mL of HPLC-grade methanol, filtered (0.22 µm polytetrafluoroethylene filter), and transferred into a vial for automatic sampling, using 5 µL for UHPLC. A UHPLC (Nexera, Shimadzu Corporation, Milan, Italy) equipped with a Zorbax ODS column (25 cm × 4.6 mm, 5 µm; Agilent Technologies, Santa Clara, CA, USA) and a Shimadzu spectrofluorometric detector (RF-20AXS, Shimadzu Corporation, Milan, Italy) (excitation wavelength of 295 nm and emission wavelength of 330 nm) were used. The UHPLC system was controlled by LabSolutions software. The quantity of sample with methanol injected was 10 µL and the analytes were identified by comparing the retention times with those of pure standards.

In samples of muscle, the analytical procedures described by AOAC [16] were followed to evaluate moisture (method 950.46), crude fat (method 991.36), ash (method 920.153), and crude protein (method 984.13) contents.

### 2.2. Antioxidant Vitamins and Fatty Acid Profile Determination in Meat

Antioxidant vitamins in muscle samples were determined as described by Rufino-Moya et al. [21] using the UHPLC described above, where the chromatographic conditions were the same as described for the feed samples.

The fatty acid composition was evaluated in the total lipids extracted following the procedures described by Folch et al. [22], where, from 5 g of samples, fat was extracted using chloroform/methanol (2:1, *v*/*v*). Subsequently, fatty acids were converted into methyl esters using hexane (1 mL) and 2 N methanolic KOH (0.05 mL) [23], with C9:0 as an internal standard. Fatty acid methyl esters were evaluated using a Varian gas chromatograph (CP 3900, Mitchell Drive Walnut Creek, CA, USequipped with a capillary column 100 m long, 25 mm i.d., film thickness 0.25 μm, CP-Sil 88 Agilent J&W). One μL of sample was injected, carried by a helium flow of 0.7 mL/min. The gas chromatograph conditions were as follows: the temperature of the FID detector was set at 260 °C; the temperature of the split–splitless injector was 220 °C, the injection rate was 120 mL/min; and the temperature program of the column was 140 °C at 4 min and a subsequent increase to 220 °C at 4 °C/min. A standard FAME mix component (Supelco Inc., Bellefont, PA, USA) was used to identify each fatty acid (expressed as mg/100 g of total fatty acids).

### 2.3. Lipid Oxidation

For monitoring meat oxidative stability, three slices (2 cm thick) of meat were stored at 4 °C (covered with PVC film) for 2 h (day 0), 4, and 7 days. Lipid oxidation was evaluated by measuring thiobarbituric acid reactive substances (TBARS) at each day of storage [24]. In brief, 2.5 g of meat samples was homogenized (in a cold-water bath) with 12.5 mL of distilled water, mixed with 10% (*w*/*v*) trichloroacetic acid (12.5 mL) and filtered. Subsequently, 4 mL of filtrate was mixed with 1 mL of 0.06 M aqueous thiobarbituric acid and incubated in a water bath at 80 °C for 90 min. Using a UV-1800 Shimadzu spectrophotometer (Shimadzu Corporation, Milan, Italy), the absorbance of the samples was measured at 532 nm. The assay was calibrated using solutions of known concentrations of 1,1,3,3,-tetra-ethoxypropane in distilled water ranging from 5 to 65 nmoles/4 mL. Results were expressed as mg of malonaldehyde (MDA)/kg of meat.

### 2.4. Statistical Analysis

The software used for statistical analyses was Minitab (version 19, Minitab Inc., State College, PA, USA). The individual animal was the statistical unit. A one-way ANOVA was used to analyze the data of final weight, carcass weight, dressing yield, DMI, ADG, FCR, chemical composition, and intramuscular FA composition of meat. A mixed model ANOVA for repeated measures was used to study the data of TBARS, considering diet, time of storage, and their interaction as fixed factors, while the individual animal was included as a random factor.

Using Tukey’s multiple comparison test, differences between means were evaluated. Significance was considered at *p* ≤ 0.05, trends toward significance when 0.05 < *p* ≤ 0.10, and the Tukey test was performed for multiple comparisons.

## 3. Results

The administration of dried bergamot pulp did not significantly influence the final body weight, feed conversion ratio (FCR), average daily gain (ADG), carcass weight and dressing yield, while dry matter intake (DMI) was higher (*p* < 0.05) for rabbits from the DBP group compared to the control one (Table 2).

No significant differences between treatments were also observed for crude protein, moisture, ether extract, and ash contents in meat. Conversely, the α-tocopherol level was higher (*p* < 0.01) in meat from rabbits supplemented with dried bergamot pulp compared to meat from control rabbits.

Table 3 shows the data on the meat fatty acid composition. Supplementation of 10% dried bergamot pulp in the rabbit diet did not influence the accumulation of IMF (*p* = 0.190) in meat (Table 3).

No significant differences were observed for the total contents of saturated fatty acids (SFAs), monounsaturated fatty acids (MUFAs), and polyunsaturated fatty acids (PUFAs).

Among the individual PUFAs, the concentrations of α-linolenic acid (ALA, C18:3 n-3) and eicosapentaenoic acid (EPA, C20:5 n-3) in muscle increased (*p* < 0.01 and *p* = 0.021, respectively) after integrating dried bergamot pulp into the diet, leading to a higher level of the total content of ω-3 fatty acids (*p* < 0.01) compared to the control treatment. Consequently, the n-6 to n-3 ratio in the meat from the DBP group was lower (*p* < 0.05) than in the meat from the control group.

**Figure 1:**
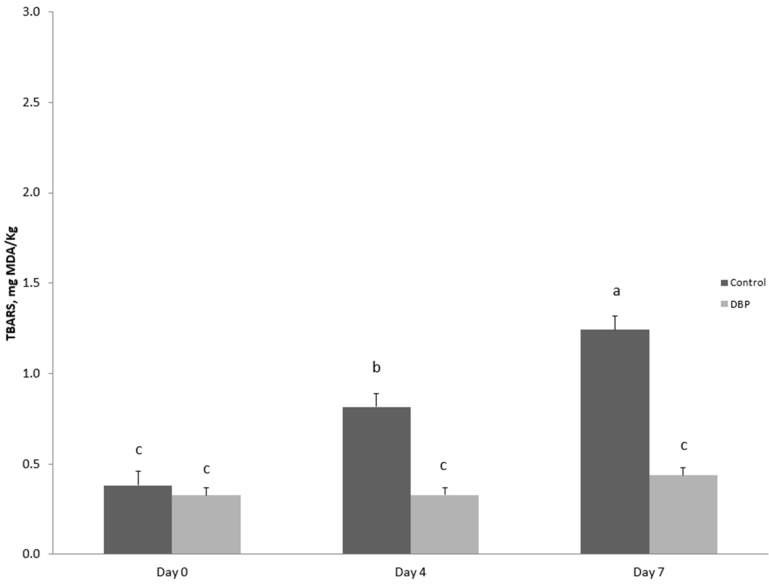
Effects of storage time and dietary treatment on TBARS values evaluated in meat slices during refrigerated storage. Dietary treatment: a basal diet (control) or the basal diet in which 5% of barley grain and 5% of maize grain was replaced by 10% of dried bergamot pulp (DBP). Values presented are estimated least squares means and standard error bars. a, b, c Values with different superscripts are significantly different (*p* ≤ 0.05). The effect of the diet was *p* < 0.001, the effect of time was *p* < 0.001 and the effect of the interaction was *p* < 0.001.

Meat oxidative stability data are shown in Figure 1. The administration of dried bergamot pulp led to a reduction in lipid oxidation (*p* < 0.001) in the meat during the 7 days of storage, with a significant diet × time interaction (*p* < 0.001). Specifically, while TBARS values in the meat of rabbits fed the basal diet increased already after 4 days of storage compared to the first day of monitoring (*p* < 0.001), increasing further on the 7th day of storage (*p* < 0.001), TBARS values remained stable in the meat of rabbits fed the DBP diet for all days of observation.

## 4. Discussion

Although, in recent years, several studies have shown the effects of using the by-products of bergamot processing on the meat of small ruminants [9,10] and pigs [11,25,26], to the best of our knowledge, no studies investigated the use of dried bergamot pulp in the rabbit diet. However, some scientific research aimed to study the effects of by-products from the industrial processing of other citrus fruits, such as oranges, on the rabbit’s diet [27,28,29].

In this trial, the dietary administration of dried bergamot pulp did not influence the main performance parameters. As shown in Table 2, the final weight and the ADG of the animals, as well as carcass weight, were comparable between treatments. This suggests that energy value of bergamot pulp is comparable to those of the grain replaced. Even when there is no information available about of feeding value of bergamot pulp for rabbits, other reports are available on the feeding value of other citrus by-products. In this sense, data reported by Zeweil et al. [30] showed that including dried orange pulp instead of barley in the rabbit diets did not affect the final body weight and carcass traits as compared to the control group. Similar results were observed in the study by Varela et al. [29], where treatment with 10% citrus pulp showed no differences (*p* > 0.05) in the performance parameters evaluated, while a higher weight gain (*p* < 0.05) was observed when increasing the integration of citrus pulp up to 20%, without any effects on carcass yield, than the control group. Even Lu et al. [28], investigating citrus pulp integration in the daily feed of rabbits, found that citrus pulp could be used in the rabbit diet at up to 21%with no adverse effects on growth performance, as also observed by De Maria et al. [31] in growing rabbits fed a diet containing 20% of dehydrated citrus pulp. Some authors [32] underline that the addition of citrus pulp in rabbit diets improved the DM and fiber digestibility, data which could explain the improvement in growth performance observed in several experimental trials. Even Ibrahim et al. [9] observed improved nutrient digestibility by integrating lemon and orange pulp into the rabbit’s diet. However, even if only numerically, the animals in the DBP group had a final weight that was approximately 10% greater than rabbits in the control group, a result which therefore could depend on better digestibility of the fiber and DM of the DBP diet.

To make healthier products, both from polygastric and monogastric animals, one of the main objectives is to find strategies to increase n-3 fatty acids and reduce saturated fatty acids [33]. Several factors can influence the fatty acid profile of rabbit meat, such as age, breed, sex, and type of tissue, but the diet received by the animal is the factor that has the greatest impact. The integration of dried bergamot pulp has led to a change in the fatty acid profile of the meat, leading to an increase in those fatty acids considered beneficial for human health. Indeed, the meat of the DBP group had significantly higher levels of linolenic acid, EPA, and total n-3 fatty acids. This situation led to an improvement in the n-6/n-3 ratio. The higher proportion of α-linolenic in meat from rabbits fed DBP than in meat from the control group agrees with the greatest intake of this fatty acid from animals fed the bergamot-supplemented diets, considering the higher level of α-linolenic acid in DBP diet than in control diet. Considering the numerous biological effects of n-3 fatty acids, especially long-chain n-3 PUFAs, which are thought to be beneficial for human health, these findings are relevant. However, the contribution of dried bergamot pulp supplementation to increased levels of linolenic acid and long-chain n-3 PUFAs in rabbit meat was small, considering that the estimated adequate intake for humans is 2.22 g/day for linolenic acid and 0.65 g/day for DHA + EPA [34].

For the growth performance of the animals, it was possible to compare the data of this experimental trial with data present in the literature, at least on experimental trials carried out using citrus pulp in general; as regards the acid profile, it is very difficult to compare our results with those of other studies since few articles are available on this topic in rabbits. Similar results were observed in our previous studies on small ruminants [9] or pigs [11], where the animals were fed a diet supplemented with bergamot by-products.

One of the most important qualitative characteristics of meat that influences its shelf-life and acceptability (sensory attributes) over time is oxidative stability. Among the many oxidative processes in meat, in this study, we focused on the oxidation of meat lipids since they can directly affect the consumer through the development of off flavors.

The diet received by the animal during growth is the variable that most influences the progress of lipid oxidation in meat during storage. The PUFA content and the presence of antioxidant compounds play fundamental roles in the shelf life of meat, and their presence strongly depends on the diet supplied. While PUFAs make meat more susceptible to lipid oxidation [35], leading to the production of conjugated dienes and hydroperoxides following their reaction with oxygen and ultimately resulting in the accumulation of volatile molecules such as aldehydes and ketones, the presence of antioxidant compounds protects it from oxidation, improving the shelf-life [36].

In this trial, integrating dried bergamot pulp into a rabbit diet improved meat oxidative stability through storage time. This result was probably influenced by the higher quantity of α-tocopherol in the meat of the DBP group compared to the meat of the control group. Lu et al. [28] observed higher (*p* < 0.001) hepatic T-AOC (total antioxidant capacity) activities in rabbits whose diet was supplemented with dried citrus pulp than in rabbits from the control group. The data are similar to those of the experimental trial by Hassan et al. [37] on rabbits fed an orange peel-supplemented diet, a by-product that led to an increase in SOD (superoxide dismutase), an enzymatic antioxidant, and T-AOC values in plasma. In our recent experimental trial [9] on lambs supplemented with fresh bergamot pulp, we found greater oxidative stability of the meat following the integration of this by-product in the diet at up to 20% compared to the meat of the control group.

As stated above, in this study, despite the amounts of α-tocopherol being similar in the experimental diets, the meat of the DBP group had the highest amount of it. We could hypothesize that bergamot pulp may offer additional antioxidant effects, which could be due to the presence of antioxidant compounds other than tocopherols. Bergamot fruit contains a very high amount of flavonoids, mainly in the peel, in particular naringin and neosperidin [38]. As emphasized by different authors [36,39], the phenolic compounds, which had greater contents in the DBP diet than in the control one, could have facilitated the absorption of vitamin E, indirectly preserving it during digestion thanks to their antioxidant activity. Several studies report that phenol compounds have different effects, including antibacterial, anti-inflammatory, immunostimulatory, and antioxidant effects [40,41]. A very high number of different flavonoids have been found in citrus fruits [12,42], the absorption of which has been observed in the small intestine [43]. Furthermore, Mandalari et al. [12] state that many of these compounds are present at higher levels in bergamot than in other citrus fruits.

Regarding a possible direct effect of phenolic compounds on the antioxidant capacity of meat, the general opinion is that it is improvable, especially for tannins, due to their low bioavailability and absorption efficiency in animals. These pinions are in agreement with the study by Surai [44], who observed a generally low absorption of polyphenols/flavonoids in chicken. However, some researchers [45] have observed higher TEAC and FRAP values in meat, indicating greater antioxidant activity, following supplementation of quebracho tannins in the lambs’ diet.

## 5. Conclusions

To date, this is the first study aimed at evaluating the effect of feeding rabbits dried bergamot pulp on animal performances and the fatty acid composition, and oxidative stability of meat. Dried bergamot pulp included at up to 10% when replacing cereal grains did not affect the intake, performance, and carcass yield of growing–finishing rabbits. The results showed that the contents of some of the fatty acids with positive health effects, such as α-linolenic acid and eicosapentaenoic acid, increased in rabbit meat following the integration of dried bergamot pulp into the diet.

The administration of dried bergamot pulp also improved the oxidative stability of meat. In conclusion, the integration of dried bergamot pulp at up to 10% in the rabbit diet could represent a valid strategy to naturally improve some important qualitative aspects of meat and to promote the exploitation of an easily available feed resource, especially in areas of citrus fruit processing, such as Mediterranean areas.

## Figures and Tables

**Table 1:** Ingredients (% on a DM basis) and chemical composition of the experimental diets (pelleted form).

	Dried Bergamot Pulp	Control Diet	DBP Diet
Barley grain		10	5
Maize grain		10	5
Wheat bran		28	28
Soybean meal		10	10
Alfalfa meal		40	40
Dried bergamot pulp			10
Vitamin mineral premix ^1^		2	2
Chemical composition			
Dry matter (DM) g/kg wet weight	887	889	891
Crude protein g/kg DM	57.6	156	154
Ether extract g/kg DM	14.1	25.4	25.6
Ash g/kg DM	51.2	38.9	40.4
NDF g/kg DM	373	325	340
Total phenolic compounds (g TAe ^2^/kg DM)	13.2	4.12	5.02
Total tannin compounds (g Tae ^2^/kg DM)	2.01	0.82	1.68
Tocopherols, μg/g dry matter			
α-Tocopherol	50.3	52.4	48.2
γ-Tocopherol	5.91	6.87	6.19
Individual fatty acids (g/100 g of total fatty acid)			
C10:0	0.07		
C12:0	0.14		0.03
C14:0	0.29	0.12	0.19
C16:0	19.1	15.1	15.6
C16:1	0.56	0.15	0.17
C18:0	3.67	3.96	3.87
C18:1 n-9	26.1	36.4	32.1
C18:2 n-6	30.1	26.1	26.8
C18:3 n-3	9.12	1.49	3.32

^1^ The mineral vitamin premix consisted of vitamin A, 6750 UI; vitamin D3, 1000 UI; vitamin E, 2 mg; vitamin B12, 0.01 mg; vitamin B1, 1 mg; folic acid, 0.2 mg; D-pantotenic acid, 5 mg; Co, 0.05 mg; Mn, 12.5 mg; Zn, 15 mg; and Mo, 0.5 mg; ^2^ tannic acid equivalents.

**Table 2:** Animal performances in vivo and muscle chemical composition (g/100 g wet weight).

Dietary Treatment ^1^				
	Control	DBP	SEM ^6^	*p* Value
Final BW ^2^, g	2828	3080	91.1	0.176
Carcass weight, g	1651	1830	62.3	0.156
Dressing yield (%)	58.4	59.4	6.52	0.146
Total DMI ^3^, g/d	154	175	4.84	0.045
ADG ^4^, g/d	35	39	2.01	0.103
FCR ^5^, g DMI ^3^/g ADG ^4^	4.4	4.5	0.23	0.283
*Tocopherols and Colesterol, µg/g muscle*				
α-Tocopherol	0.24	2.86	0.365	0.01
γ-Tocopherol	0.17	0.18	0.007	0.128
Cholesterol	1.34	1.27	0.026	0.214
*Chemical composition*				
Moisture	75.2	74.5	0.191	0.771
Crude protein	22.5	22.7	0.189	0.561
Ether extract	1.60	1.66	0.138	0.420
Ash	2.26	2.28	0.132	0.519

^1^ Dietary treatment: a basal diet (control) or the basal diet in which 5% of barley grain and 5% of maize grain was replaced by 10% of dried bergamot pulp (DBP). ^2^ BW = body weight; ^3^ DMI = dry matter intake; ^4^ ADG = average daily gain; ^5^ FCR = feed conversion ratio; ^6^ SEM = standard error of the mean. Significance was considered at *p* ≤ 0.05.

**Table 3:** Effects of the dietary treatments on the fatty acid composition of muscle (mg/100 g of meat).

Dietary Treatment ^1^				
Item	Control	DBP	SEM ^3^	*p* Value
intramuscular fat, mg/100 g of muscle	1600	1484	156	0.190
C12:0	1.28	1.34	0.165	0.621
C14:0	27.4	24.6	2.542	0.201
C14:1 *cis*-9	0.96	0.89	0.154	0.231
C16:0	333	309	39.7	0.192
C17:0	4.96	3.86	0.821	0.101
C16:1 *cis-9*	21.8	19.1	2.312	0.341
C18:0	261	224	25.1	0.154
C18:1 *cis-9*	579	516	75.2	0.213
C18:1 *trans-11*	15.7	21.1	2.39	0.089
C18:1 *trans-9*	5.12	5.94	0.892	0.221
C18:2 *cis-9*, *cis-12* LA ^2^	162	147	19.6	0.321
C18:3 n-3 ALA^2^	6.56	20.9	0.971	0.001
C20:2 n-6	10.4	9.50	0.421	0.564
C20:3 n-6	1.12	1.04	0.152	0.476
C20:3 n-3	3.04	5.94	0.721	0.078
C20:4 n-6	67.7	52.7	7.21	0.092
C20:5 n-3 EPA ^2^	3.20	6.86	0.781	0.021
C22:6 n-3 DHA ^2^	3.20	4.01	0.732	0.213
∑ n-3	16.1	37.7	2.872	0.001
∑ n-6	242	211	21.34	0.121
n-6/n-3	15.1	5.60	0.972	0.023
∑ SFAs ^2^	628	563	93.2	0.365
∑ MUFAs ^2^	622	563	89.3	0.321
∑ PUFAs ^2^	257	248	26.1	0.134

^1^ Dietary treatment: a basal diet (control) or the basal diet in which 5% of barley grain and 5% of maize grain was replaced by 10% of dried bergamot pulp (DBP). ^2^ LA: linoleic acid; ALA: α-linolenic acid; EPA: eicosapentaenoic acid; DHA: docosahexaenoic acid; SFAs: saturated fatty acids; MUFAs: monounsaturated fatty acids; PUFAs: polyunsaturated fatty acids. ^3^ SEM = standard error of the mean. Significance was considered at ≤0.05.

## Data Availability

The original contributions presented in the study are included in the article, further inquiries can be directed to the corresponding author.
